# The first complete mitochondrial DNA of *Tenuidactylus dadunensis* (Squamata: Gekkonidae) and its phylogeny

**DOI:** 10.1080/23802359.2024.2333566

**Published:** 2024-04-02

**Authors:** Qian-Ru Liang, Lei Shi

**Affiliations:** Xinjiang Key Laboratory for Ecological Adaptation and Evolution of Extreme Environment Biology, College of Life Sciences, Xinjiang Agricultural University, Urumqi, China

**Keywords:** Gekkonidae, *Tenuidactylus dadunensis*, mitogenome, phylogenetics relationship

## Abstract

The complete mitochondrial DNA (mtDNA) of *Tenuidactylus dadunensis* (Squamata: Gekkonidae) was described by using next-generation sequencing. The total length of mtDNA was 16,893 bp, which contained 13 PCGs (*COX1-3*, *ND1-6*, *ND4L*, *ATP6*, *ATP8*, and *CYTB*), 22 transfer RNA(tRNA) genes, 2 ribosomal RNA (rRNA) genes, and a control region (D-loop). The Bayesian inference tree showed that *T. dadunensis* was included in Gekkonidae and was a sister taxon to *Cyrtopodion scabrum*. The complete mtDNA of *T. dadunensis* will be an important genetic resource to the studies of conservation and research of geckonids.

## Introduction

**Figure 1.**
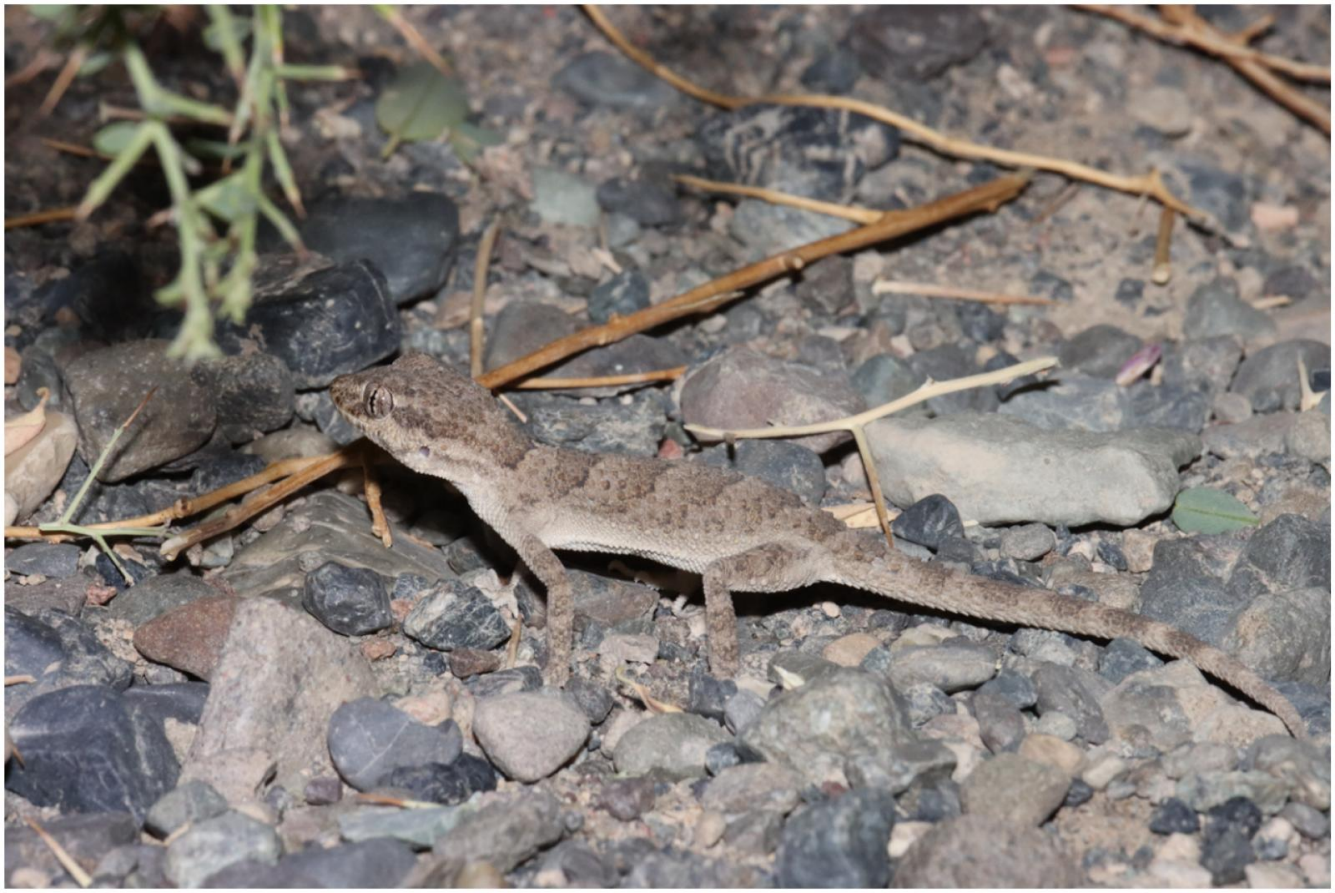
Photograph of *Tenuidactylus dadunensis* (photo credit: Lei Shi, used with permission). Specimen for our study were collected in Turpan by Weizheng Gao, Tao Wang and Bo Ma.

*Tenuidactylus dadunensis* (Shi and Zhao 2011) (ICUN: DD) (Figure 1) is a species of *Tenuidactylus* in the family Gekkoidea which is endemic to Xinjiang, China. Shi and Zhao (2011) were the first to discover this species in Turpan Basin, western China and named it *Cyrtopodion dadunense*. Based on morphological analysis, they concluded that this species was significantly different from two other species of the same genus (*Cyrtopodion elongatum* and *Cyrtopodion russowi*) distributed in Xinjiang. The phylogeny and classification of the Palearctic naked-toed geckos (*Cyrtodactylus seso lato*) was subsequently revised by Bauer et al. (2013), which supported that three subgenus of *Cyrtopodion,* including *Cyrtopodion, Tenuidactylus* and *Mediodactylus* should be arised to valid genus. Meanwhile, Bauer et al. (2013) tentatively place *Cyrtopodion dadunense* in this genus, as *Tenuidactylus daduensis*. Kai et al. (2020) followed Bauer et al. (2013) in their update of the Chinese amphibian and reptile list. Since then, no further studies on *T. daduensis* have been reported. *Tenuidactylus* is Palearctic thin-toed gecko, which has a total of eight species, including *Tenuidactylus bogdanovi, T. caspius, T. dadunensis, T. elongatus, T. fedtschenkoi, T. longipes, T. turcmenicus, T. voraginosus* (Uetz and Stylianou 2018), no more molecular infomation reported untill now. In this study, we used Illumina NovaSeq 6000 sequencing technology for paired-end sequencing of the sample DNA to obtain the complete mitochondrial genome of *T. daduensis*. This is the first complete mitochondrial sequence of *T. daduensis* and the first complete sequence of genus *Tenuidactylus*, which will provide data support for the phylogenetic studies of Gekkonidae in the future.

## Materials and methods

Samples of *T. dadunensis* were collected on 28 June 2023 in Turpan City (42.85 N, 88.87E), Xinjiang UygurAutonomous Region, China. The specimen (NO: THAT5702-9) was identified by Lei Shi. We based our identification on the description of *T. dadunensis* by Shi and Zhao (2011): nostril bordered by rostral, first supralabial, one supranasal, and two subequal postnasals; back tubercles arranged in regular longitudinal rows; 23–26 ventral scales across midabdomen; 97–108 scales along the ventrum of body from postmental to cloaca; 18–22 subdigital lamellae beneath fourth toe; 8–10 precloacal pores in males; caudal tubercles broadly in contact laterally with each other; a single row of transversally enlarged subcaudals; dorsal bands in waving shape, much thinner than interspaces. We cut a portion of tissue from the tail, and the specimen was preserved in the Animal Herbarium of the College of Life Sciences, Xinjiang Agricultural University, Xinjiang, China (Lei Shi, leis@xjau.edu.cn).

Total genomic DNA was extracted from the adductor muscle tissue using a DNeasy tissue kit (Qiagen, Beijing, China) following the manufacturer’s protocols. After DNA isolation, 1 μg of purified DNA was fragmented to ∼500 bp using the Covaris M220 system, used to construct short-insert libraries according to the manufacturer’s instructions (TruSeq™Nano DNA Sample Prep Kit, Illumina), and then sequenced on an Illumina NovaSeq 6000 platform (Modi et al. 2021) (BIOZERON Co., Ltd, Shanghai, China). The mitochondrial genome was reconstructed using a combination of denovo and reference-guided assemblies, and the following three steps were used to assemble the mitogenome. First, the filtered reads were assembled into contigs using GetOrganelle v1.7.5 (Jin et al. 2020), potential mitochondrial contigs were extracted by aligning against the NCBI mitogenome database. Second, the potential mitochondrial contigs were aligned to the reference mitogenomes using BLAST v 2.8.1+, and aligned contigs (>80% query coverage) were ordered and connected manually according to the reference mitogenomes. Finally, MUMmer 3.23 (Kurtz et al. 2004) was used to check whether these contigs were circular. Based on the above assembly steps, we obtained a circle of *T. dadunensis* mitogenome. We used GetOrganelle v1.7.5 software (https://github.com/Kinggerm/GetOrganelle) (Jin et al. 2020) for mitochondrial genome assembly. The software cycles through the target reads using the seed database and then calls SPAdes to assemble the genome. We performed reads mapping with Bowtie2 (Langdon 2015) and calculated the reads coverage of the mitochondrial region by SAMtools depth (Li et al. 2009). We compared the sequencing reads to the assembly results to get a depth of coverage map of the whole genome sequence (Supplementary Figure S1). The mitochondrion genes were annotated using the online MITOS tool (Bernt et al. 2013), using default parameters to predict protein coding genes, transfer RNA (tRNA) genes, and ribosome RNA (rRNA) genes.

DNA sequences were aligned using ClustalW (Thompson et al. 1994) implemented in MEGA7.0 (Kumar et al. 2016). In order to clarify the phylogenetic position of the entire mitochondrial sequence of *T. dadunensis*, we obtained 27 complete mitochondrial genome sequences from the GenBank database from Gekkonidae, Phyllodactylidae, Sphaerodactylidae and Eublepharidae, respectively (Table 1) (Ma et al. 2020). We selected all available mitogenomes under these taxonomic designation to determine a clear phylogenetic position for *T. dadunensis* with a relatively complete phylogenetic tree. We used Partitionfinder-2.1.1 (Lanfear et al. 2017) to estimate the best model for the molecular dataset using the Akaike information criterion. We used Bayesian Inference (BI) to construct phylogenetic relationships for *T. dadunensis*, with the species of Eublepharidae as the outgroup. We then used MrBayes 3.2.2 (Ronquist et al. 2012) to perform Bayesian inference (BI). We used the Markov chain Monte Carlo (MCMC) theory approach in BI, running 8,000,000 generations, drawing one sample every 1000, running four chains per analysis, and removing the top 25% of the data with the other parameters as the default. The final results were checked using Tracer 1.7.2 (Rambaut et al. 2018) and had effective sample size (ESS) values > 200 for all parameter values. Finally, a phylogenetic tree was plotted using ITOLV6 (Letunic and Bork 2021). We considered an a posteriori probability (BPP) value of > 0.95 as strong support for monophyly.

**Table 1.** Mitochondrial genome sequences with GenBank accession numbers used in this study.

Species	Size (bp)	GenBank NO.	source
*Tenuidactylus dadunensis*	16.893	OR537229	this study
*Gekko gecko*	16.435	AY282753	Zhou et al. (2006)
*Gekko swinhonis*	16.818	JQ906550	Li et al. (2013)
*Cyrtopodion scabrum*	16.994	AB661665	–
*Cyrtodactylus louisiadensis*	17.594	AB606970	–
*Cnemaspis limi*	16.680	HQ896026	Yan et al. (2014)
*Gehyra marginata*	16.588	AB661662	–
*Lepidodactylus lugubris*	16.762	AB738945	Kumazawa et al. (2014)
*Pachydactylus punctatus*	17.475	AB612270	–
*Paroedura picta*	17.220	KR149293	Starostová and Musilová (2016)
*Phelsuma guimbeaui*	17.533	AB661664	Kumazawa et al. (2014)
*Tropiocolotes tripolitanus*	20.248	AB661661	Kumazawa et al. (2014)
*Stenodactylus petrii*	18.672	AB738952	Kumazawa et al. (2014)
*Uroplatus fimbriatus*	16.780	AB612276	Kumazawa et al. (2014)
*Hemidactylus frenatus*	16.891	GQ245970	Li et al. (2013)
*Hemidactylus ulii*	16.901	OL689330	Šmíd et al. (2023)
*Hemidactylus mandebensis*	16.830	OL689329	Šmíd et al. (2023)
*Phyllodactylus unctus*	16.881	HQ896027	–
*Tarentola mauritanica*	15.327	JQ425060	Rato et al. (2013)
*Teratoscincus przewalskii*	17.184	OL471044	–
*Teratoscincus roborowskii*	16.693	MT107158	Ma et al. (2020)
*Teratoscincus microlepis*	16.995	AB612275	–
*Coleonyx variegatus*	17.110	AB114446	Kumazawa (2007)
*Eublepharis macularius*	17.460	OQ420358	Pinto et al. (2023)
*Goniurosaurus varius*	17.778	OQ992199	Zhou et al. (2023)
*Goniurosaurus luii*	16.519	KM455054	–
*Hemitheconyx caudicinctus*	17.043	AB610502	Jonniaux et al. (2012)

## Results

**Figure 2.**
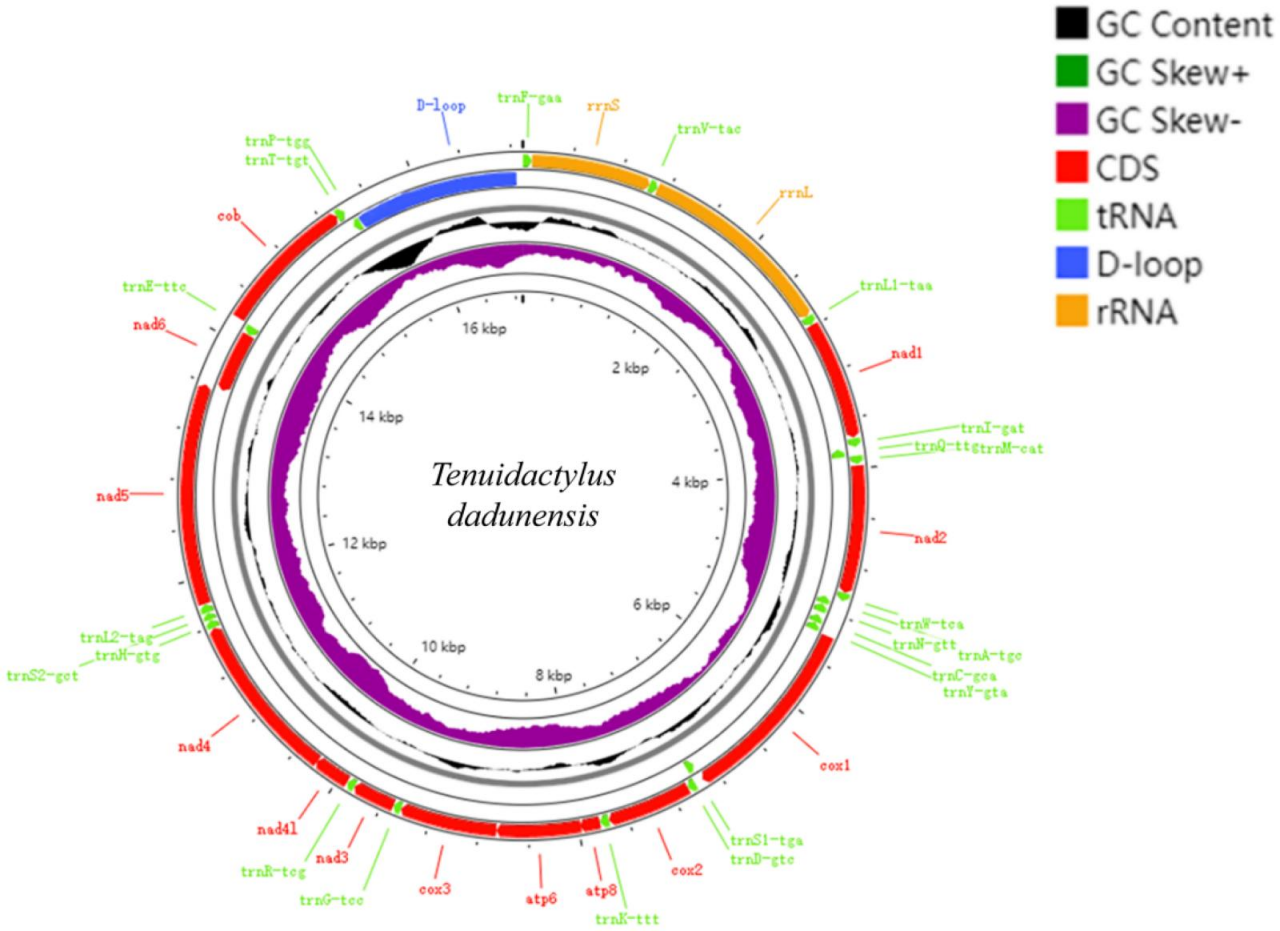
Mitochondrial genome maps for *Tenuidactylus dadunensis* (OR537229).

**Figure 3.**
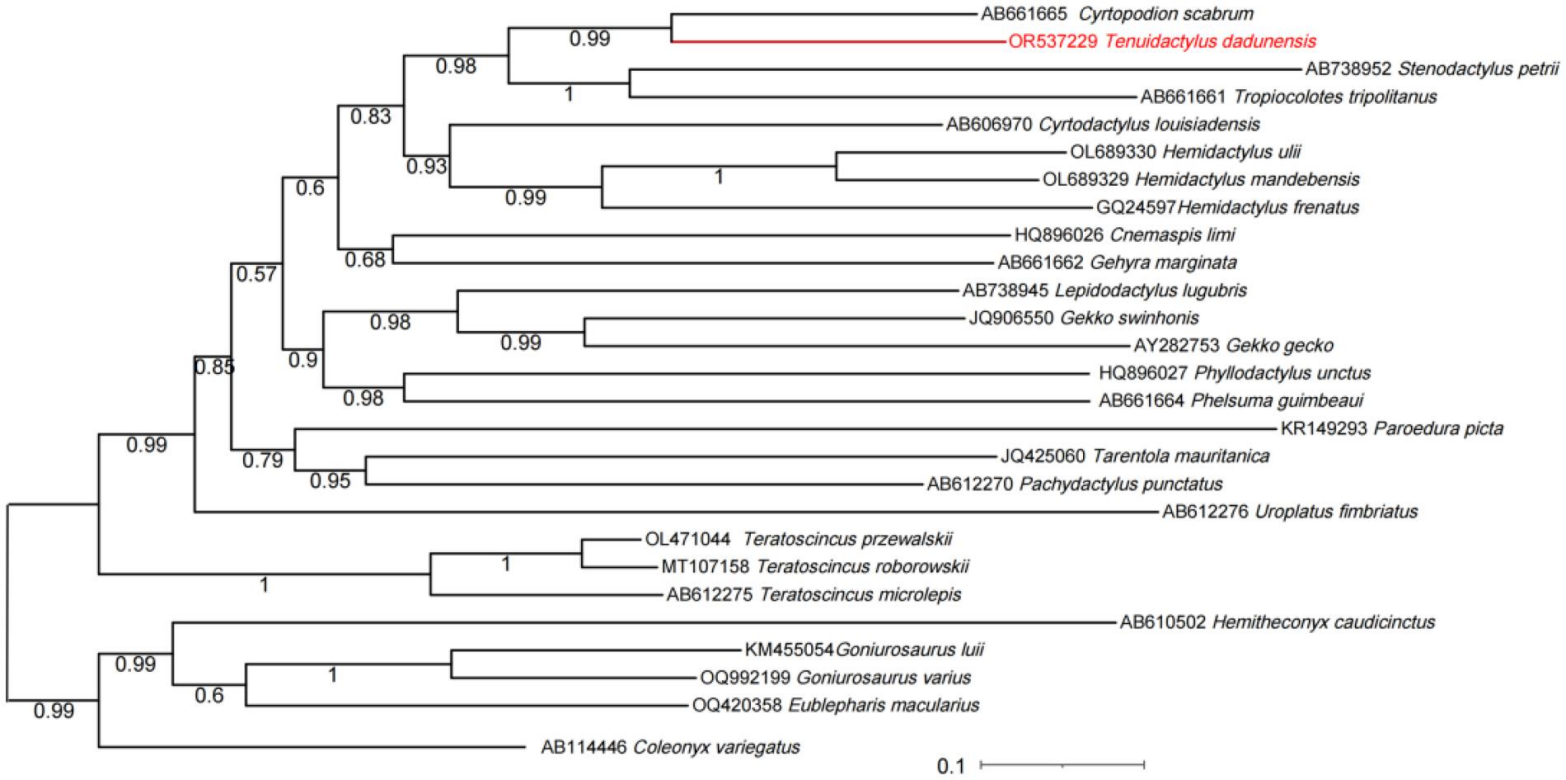
The Phylogenetic tree of *Tenuidactylus dadunensis* (OR537229) and other available mitogenomes under these taxonomic. The numbers showed between branches indicate the posteriori probabilities from Bayesian inference (BI). The GenBank accession numbers of all species are shown in the figure and the citations are in the Table 1.

The length of *T. dadunensis* complete mtDNA (GenBank accession: OR537229) was 16,893 bp, which contained 13 PCGs (*COX1-3*, *ND1-6*, *ND4L*, *ATP6*, *ATP8*, and *CYTB*), 22 *tRNA* genes, 2 *rRNA* genes, and a control region (D-loop) (Figure 2). Depth of coverage maps for whole genome sequences are shown in Supplementary Figure S1. The gene order was similar to the known mitogenomes of Gekkonidae (Zhou et al. 2006; Yan et al. 2009; Ma et al. 2020). The nucleotide composition of *T. dadunensis* was 32.68% A, 24.02% T, 28.78% C and 14.52% G. Based on the results of the Bayesian phylogenetic tree (Figure 3), we can know the location of our mitotic genome samples (*T. dadunensis*) in the the genomic dataset of Gekkonidae. The results of the Bayesian phylogenetic tree (Figure 3) show that *T. dadunensis* was clustered into the clade of genomic dataset of Gekkonidae, and was a close sister clade to *C. scabrum,* with strongly support (BI = 0.99). More mitogenomic data may be needed at a later stage to analyze and clarify the specific position of *T. dadunensis* within the Gekkonidae.

## Discussion and conclusion

In this study, mitochondrial genomes were sequenced and assembled for *T. dadunensis*. The structure of the whole mitochondrial sequence of *T. dadunensis* is similar to that of other species of the same family. The mitogenome of *T. dadunensis* was 16,893 bp in size, including 13 PCGs, 22 transfer RNA(tRNA) genes, 2 ribosomal RNA (rRNA) genes, and a control region (D-loop). The complete mtDNA of *T. dadunensis* reported in this study will play an important role in understanding the evolution and systematic biology of the family Gekkonidae. The phylogenetic results show that *T. dadunensis* is closely related to *C. scabrum*, and also show that the Gekkonidae are monophyletic. Furthermore, it will be an important genetic resource to the studies of conservation and restoration of *T. dadunensis.*

## Supplementary Material

Supplemental Material

Supplemental Material

## Data Availability

The genome sequence data that support the findings of this study are openly available in GenBank of NCBI at (https://www.ncbi.nlm.nih.gov/) under the accession OR537229. For *T. dadunensis*the associated BioProject, BioSample and SRA numbers are PRJNA1050867, SAMN37889599, SRR27167919.

## IUCN species information

*Tenuidactylus dadunensis* has most recently been assessed for The IUCN Red List of Threatened Species in 2018. *Tenuidactylus dadunensis* is listed as Data Deficient.

(https://www.iucnredlist.org/species/47755066/47755073)

The species studied comply with the International Union for Conservation of Nature (IUCN) policies research involving species at risk of extinction, the Convention on Biological Diversity and the Convention on the Trade in Endangered Species of Wild Fauna and Flora.
