# Supplementary figures and images for: The first complete mitochondrial DNA of Tenuidactylus dadunensis (Squamata: Gekkonidae) and its phylogeny
Source: Mitochondrial DNA B Resour. 2024 Apr 2;9(4):442–6. doi: 10.1080/23802359.2024.2333566 (PMC10993739; doi:10.1080/23802359.2024.2333566)

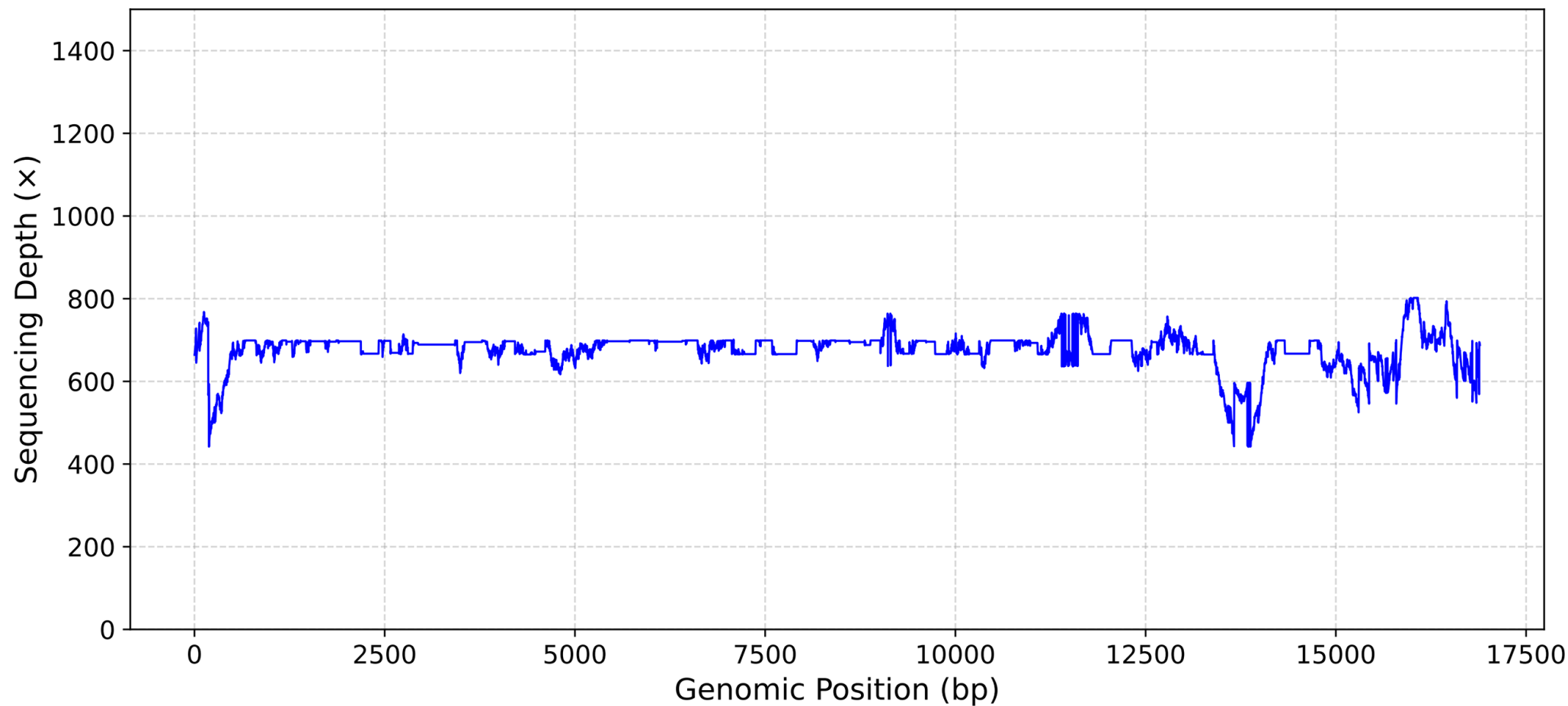

(1) Total genome length = 16,893 bp  
(3) Maximal depth = 802 x

(2) Average depth = 675.90 x  
(4) Minimal depth = 442 x

Supplement: Supplemental Material [file TMDN_A_2333566_SM2292.pdf]
