# Supplementary material for: The first complete mitochondrial DNA of Tenuidactylus dadunensis (Squamata: Gekkonidae) and its phylogeny
Source: Mitochondrial DNA B Resour. 2024 Apr 2;9(4):442–6. doi: 10.1080/23802359.2024.2333566 (PMC10993739; doi:10.1080/23802359.2024.2333566)
Supplement: Supplemental Material [file TMDN_A_2333566_SM2290.pdf]

The first complete mitochondrial DNA of *Tenuidactylus dadunensis* (Squamata: Gekkonidae) and its phylogeny

Qian-Ru Liang[a,b], Lei Shi[a,b],*

[a]College of Life Sciences, Xinjiang Agricultural University, Urumqi, Xinjiang 830052, China

[b]Xinjiang Key Laboratory for Ecological Adaptation and Evolution of Extreme Environment Biology, College of Life Sciences, Xinjiang Agricultural University

***Correspondence:** Lei Shi, College of Life Sciences, Xinjiang Agricultural University, Urumqi, Xinjiang 830052, China. Email: leis@xjau.edu.cn

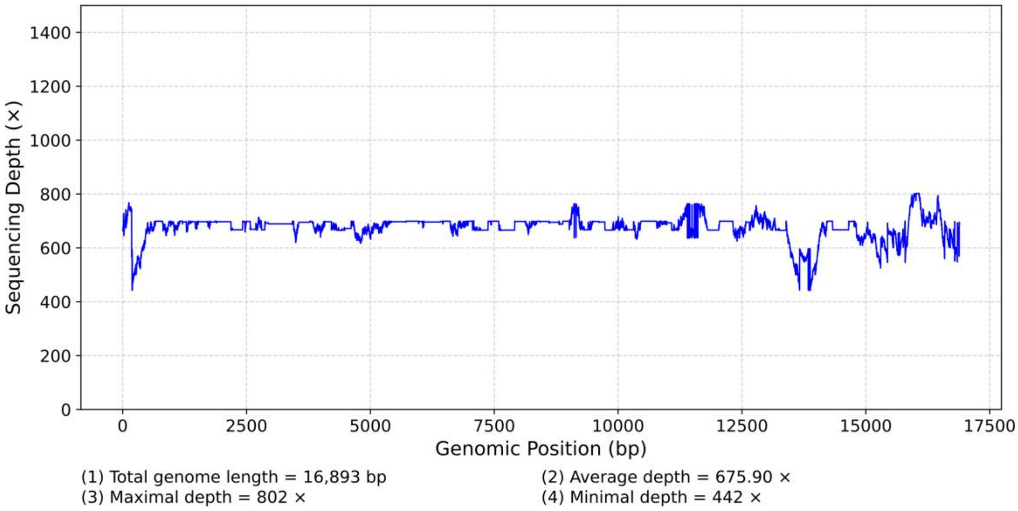

Supplementary Figure S1. Depth of coverage maps of the mitochondrial genome of *Tenuidactylus dadunensis*.
